# A rare occurrence of a steroid cell tumor of the pelvic mesentery: a case report

**DOI:** 10.1186/1752-1947-5-517

**Published:** 2011-10-18

**Authors:** Kanchan Murhekar, Robert Louis, Urmila Majhi

**Affiliations:** 1 Department of Pathology, Cancer Institute (WIA), Adyar, Chennai, Tamil Nadu, 600 020, India; 2 Department of Medical Oncology, Cancer Institute (WIA), Adyar, Chennai, Tamil Nadu, 600 020, India

## Abstract

**Introduction:**

Steroid cell tumors are microscopically characterized by abundant eosinophilic or vacuolated cytoplasm that is often positive for fat stains. These tumors could be of ovarian or ectopic adrenal origin. We present a rare case of a steroid cell tumor arising from the pelvic mesentery.

**Case presentation:**

A 31-year-old Asian woman was undergoing treatment for infertility and virilizing symptoms. She underwent laparoscopy followed by laprotomy for a suspected ovarian cyst/pelvic mass. During the laprotomy, a mass of 9 × 7 cm was detected in the pelvic mesentery.

Microscopically, the tumor showed large cells arranged predominantly in sheets with abundant granular cytoplasm and large vesicular nuclei with prominent nucleoli. The tumor was seen infiltrating surrounding adipose tissue. Immunohistochemically, the tumor cells showed strong positivity for kertain, inhibin, vimentine, melan-A, neuron-specific enolase, chromogranin, and S-100 protein and focal positivity to epithelial membrane antigen. An MIB1 index showed 4% nuclear positivity. The tumor cells were negative for calretinin, desmin, and muscle actin. Considering the clinical findings, histomorphology, and immunohistochemistry, we made the diagnosis of extraovarian and extra-adrenal steroid cell tumor (not otherwise specified) of the pelvic mesentery.

**Conclusions:**

We report an extremely rare case of an extraovarian and extra-adrenal steroid cell tumor of the pelvic mesentery. The tumor was a cause of virilizing symptoms and infertility in the patient. Surgical removal of the tumor reverted the symptoms of virilization, and the patient subsequently conceived. Steroid cell tumors should be considered in differential diagnosis among women presenting with infertility and symptoms of virilization.

## Introduction

Steroid cell tumors are rare sex cord neoplasms that account for less than 0.1% of all ovarian tumors. These tumors are composed of large round or polyhydral cells that resemble lutein, Leydig, and adrenocortical cells [1]. The majority of them produce steroids, particularly testosterone, and present with virilizing symptoms such as hirsutism, temporal balding, and amenorrhea [2-5]. They have been divided into three subtypes according to their cell of origin: stromal luteoma arising from ovarian stroma, Leydig cell tumor arising from Leydig cells in the hilus, and steroid cell tumor (not otherwise specified, or NOS) when the lineage of the tumor is unknown [1,2]. NOS tumors account for approximately half of the steroid cell tumors, occur at any age, and often are associated with virilization, although few of the tumors are also associated with estrogenic manifestations. Besides having an ovarian origin, these tumors could have an ectopic adrenal origin [1,6-8]. In this report, we present a rare case of a mesenteric steroid cell tumor.

## Case presentation

A 31-year-old Asian woman presented in our institute with a history of pelvic mass. Prior to this consultation, she was undergoing treatment for infertility. She underwent laparoscopy followed by laprotomy in a non-oncological center for a suspected right ovarian cyst/pelvic mass. During the laprotomy, a mass of 9 × 7 cm was detected in the pelvic mesentery. The tumor was hemorrhagic, dark red/brown in color, and soft to firm in consistency and was seen arising from the pelvic colonic mesentery. The adjacent bowel lumen, uterus, and both tubes and ovaries were normal. There was no evidence of adhesions, tuberculosis, or endometriosis in the pelvic peritoneum. The mass was excised.

The patient came to our institute for further management. Her ECOG performance status was grade-1 and she had no supraclavicular lymphadenopathy and there was no palpable mass or free fluid in her abdomen. The results of per-vaginal and per-rectal examinations were normal. She had been married for two years and was undergoing treatment for infertility for the previous six months when she noticed facial hairs (features of hirsutism) and a deep husky voice. Her periods were regular for the last year and a half but were reduced to spotting lasting one day every 35 to 40 days during the last six months. When she came to our institute a month and a half after the surgical excision, the facial hairs had disappeared, her periods had normalized to a regular 3/28-day cycle, and she had a normal feminine voice.

Her hemogram and chest X-ray were normal. Her serum human chorionic gonadotropin, alpha fetoprotein, and lactate dehydrogenase levels were 0.29 mIU/ml, 3 ng/mL and 458IU/L respectively. On computed tomography scan, liver, gall bladder, pancreas, spleen, and kidneys were normal. Both the adrenals as well as ovaries were normal, and no mass lesions were identified. There was no para-aortic lymphadenopathy and no free fluid in the peritoneum or pleural cavity.

**Figure 1:**
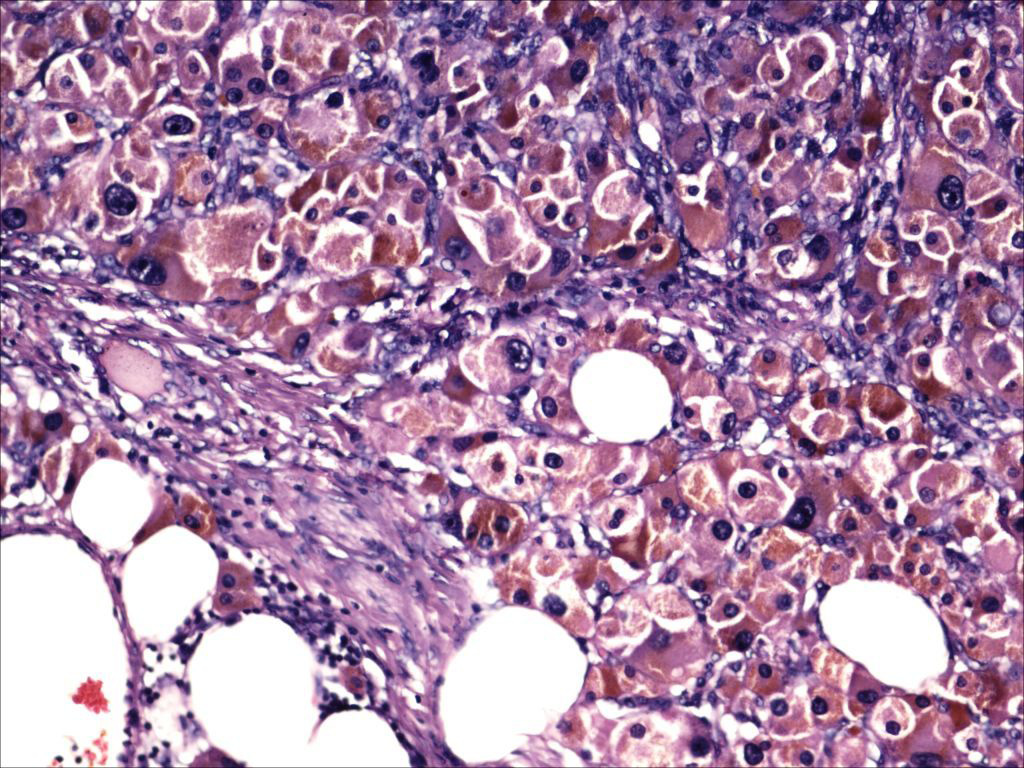
Steroid cell tumor. Stain: hematoxylin and eosin. Magnification: ×10.

**Figure 2:**
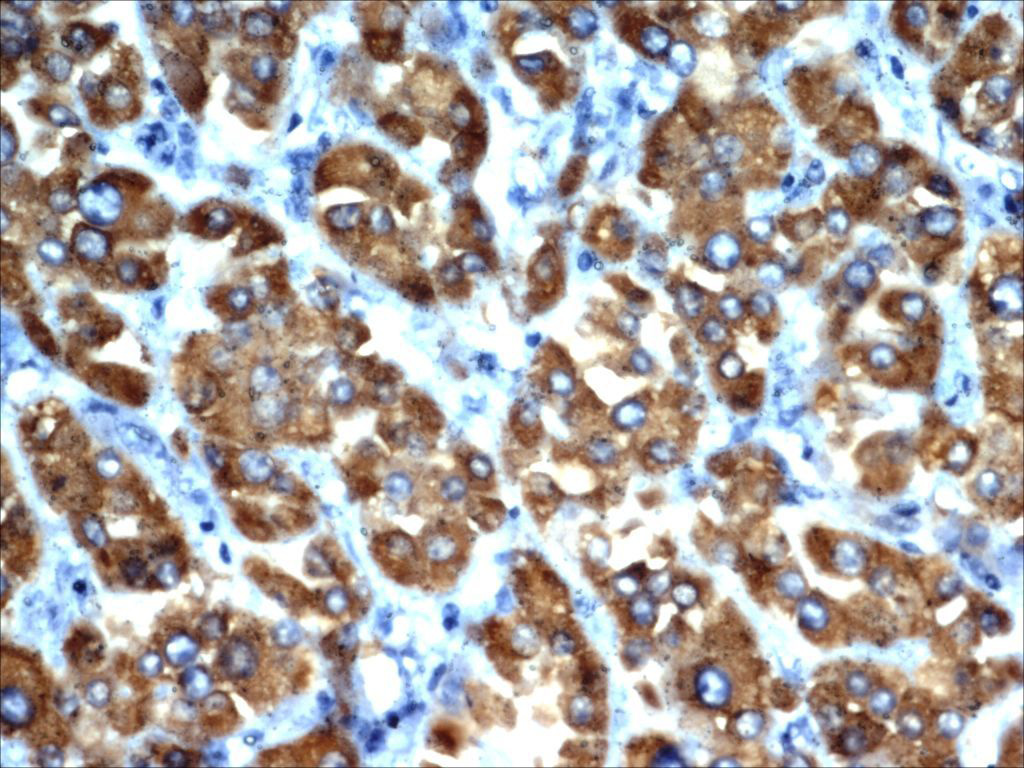
**Tumor cells showing strong positivity for inhibin.** Stain: hematoxylin and eosin. Magnification: ×10.

**Figure 3:**
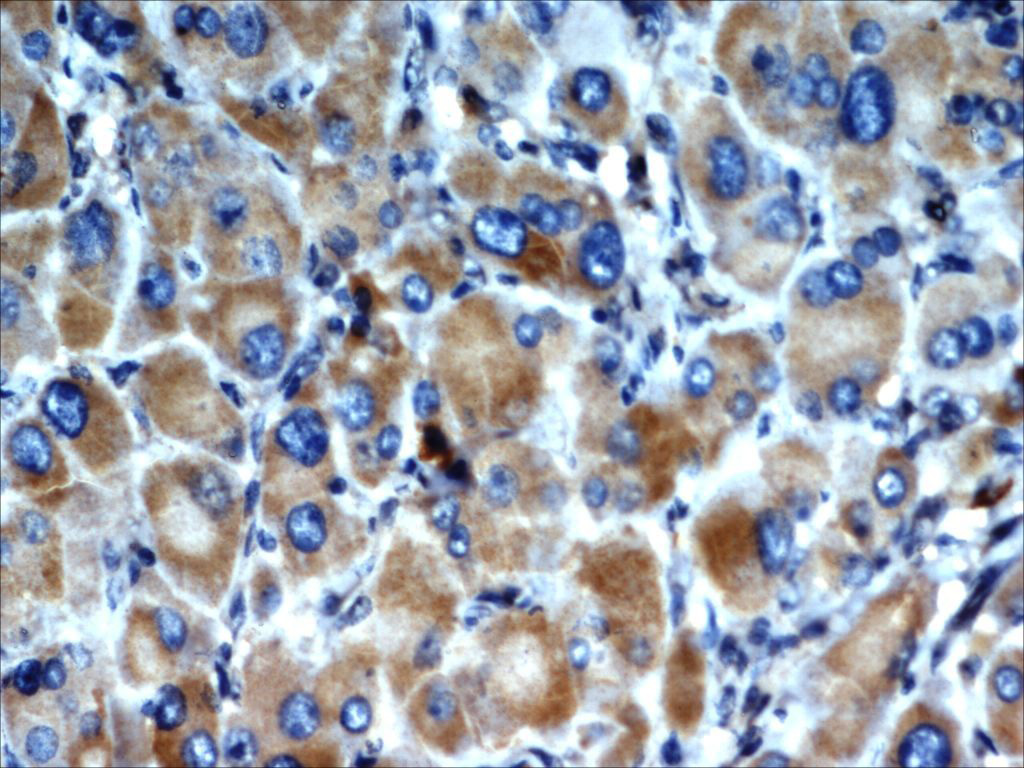
**Tumor cells showing positivity to epithelial membrane antigen**.

We reviewed the slides of the excised pelvic mass. Microscopically, the tumor showed cells arranged predominantly in sheets. The cells were large and round or polygonal with abundant granular cytoplasm (Figure 1). The nuclei were large vesicular nuclei with prominent nucleoli and showed a moderate degree of nuclear pleomorphism. The tumor was seen infiltrating surrounding adipose tissue. Immunohistochemically, the tumor cells showed strong positivity for kertain, inhibin (Figure 2), vimentine, melan-A, neuron-specific enolase, chromogranin, and S-100 protein and focal positivity to epithelial membrane antigen (EMA) (Figure 3). An MIB1 index showed 4% nuclear positivity. The tumor cells were negative for calretinin, desmin, muscle actin, and CK7. Considering the clinical findings, histomorphology, and immunohistochemistry, we made the diagnosis of extraovarian and extra-adrenal steroid cell tumor (NOS) of the pelvic mesentery.

## Discussion

The steroid cell tumors could be of ovarian or ectopic adrenal origin, whereas few cases of extraovarian and extra-adrenal origin have been reported in the literature [1,4,5,7]. However, in the majority of the cases, the exact origin of the tumor remains undecided [1].

Microscopically, steroid cell tumors are characterized by the presence of diffusely arranged cells with abundant granular eosinophilic or vacuolated cytoplasm that is often positive for fat stains. Besides these microscopic features, immunohistochemistry is very useful in diagnosing these tumors correctly.

Zhao and colleagues [9] conducted a study to find the most sensitive and robust immunohistochemical markers for different categories of sex cord tumors. A number of immunohistochemical markers were reviewed for their utility in the differential diagnosis of sex cord-stromal tumors, and inhibin and calretinin were found to be most useful in differentiating sex cord-stromal from non-sex cord-stromal tumors [9]. In an immunohistochemical study on 215 ovarian tumors, caleritinin was found to be a sensitive marker for sex cord tumors; however, it was a less specific marker than inhibin in differentiating these stromal tumors from fibrous neoplasms [10]. Most of the steroid cell tumors are positive for calretinin and inhibin [9,11,12]. However, some authors have also reported calretinin-negative steroid cell tumors [13,14].

We made the diagnosis of sex cord steroid cell tumor on the basis of microscopic pictures as well as immune reactivity to inhibin. Negative reaction to CK7 was in support of a diagnosis of sex cord tumor as these tumors are generally negative for CK7 [9,15]. Many of the sex cord tumors are also negative to EMA [9,15], although a small subset of tumors could be positive to EMA [9]. Focal positivity to EMA was reported in a case of steroid cell tumor [14]. Focal EMA positivity observed in our case is in concurrence with other reported cases [14].

Adrenocortical carcinoma and paraganglioma are the close differential diagnosis of steroid cell tumors. We ruled out the diagnosis of adrenocortical carcinoma and paraganglioma on the basis of immunohistochemical markers. Adrenocortical carcinomas are negative to EMA, keratin, and chromogranin, whereas the tumor in the present case was positive for these markers. An absence of mass lesions in bilateral adrenals further ruled out the diagnosis of adrenocortical carcinoma. Paragangliomas are negative to keratin and melan-A, whereas the tumor in the present case was positive for these markers.

Most steroid cell tumors cause virilizing syndromes. Most of these tumors are often associated with elevated levels of testosterone and androstenedione, whereas others are associated with estrogenic and progestogenic manifestations [6]. Since our patient was operated on elsewhere, we could not conduct hormonal assays. However, the clinical presentation, including the symptoms of virilization, supports the diagnosis of steroid hormone secretion. Also, within two months of the removal of the pelvic mass, symptoms of virilization disappeared.

Steroid cell tumors (NOS) also need to be differentiated from other ovarian tumors - such as the juvenile granulosa cell tumor with luteinization, lutinized thecoma, and lipid-rich Sertoli cell tumor - in which proliferation of steroid hormone-producing cells occurs as a secondary event [1,2]. A predominantly diffuse pattern in luteinized granulosa cells might suggest the diagnosis of steroid cell tumor, but the uniformity of the pattern and cytologic feature of the steroid cell tumor would be unusual for the juvenile granulosa cell tumor, which almost always contains diagnostic areas with follicles. When the lutein cells in a lutinized thecoma are extensive, a steroid cell tumor (NOS) may be simulated. Although some steroid cell tumors (NOS) might have a fibromatous component like that of thecoma, the fibromatous component in NOS tumors accounts for less than 10% of the tumor. Differentiating a lipid-rich Sertoli cell tumor with a diffuse pattern from a steroid cell tumor (NOS) depends mostly on identifying areas with a tubular pattern in the former [1,2].

The incidence of clinical malignancy among the steroid cell tumors has been documented. Hayes and Scully [3] identified five pathologic features that are highly associated with malignancy: (a) size (diameter of 7 cm or greater; 78% malignant), (b) mitoses (two or more mitoses per 10 high-power fields; 92% malignant), (c) necrosis (86% malignant), (d) hemorrhage (77% malignant), and (e) grade 2 or 3 nuclear atypia (64% malignant). The size of the tumor, presence of hemorrhage, and nuclear pleomorphism in the present case were suggestive of a malignant nature.

By extrapolation from ovarian steroid cell tumors (NOS), treatment of extraovarian steroid cell tumors is primarily surgical [4]. In the present case, the tumor was excised in a non-oncological setting. Further management issues included whether to re-excise the remnant mesentery to prevent local relapse and administer adjuvant chemotherapy to prevent distant relapse. As we were discussing these issues, our patient reported missing her period. Her urine pregnancy test was positive, and her serum beta-human chorionic gonadotropin level was 3280 mIU/mL. Our patient chose to be on regular follow-up with us and is under the care of a local obstetrician for her pregnancy.

## Conclusions

The present case was a rare report of an extraovarian and extra-adrenal steroid cell tumor of the pelvic mesentery. The tumor was a cause of virilizing symptoms and infertility in our patient. Surgical removal of the tumor reverted the symptoms of virilization, and our patient subsequently conceived. Steroid cell tumors should be considered in differential diagnosis among women presenting with infertility and symptoms of virilization.

## Abbreviations

EMA: epithelial membrane antigen; NOS: not otherwise specified;

## Consent

Written informed consent was obtained from the patient for publication of this case report and any accompanying images. A copy of the written consent is available for review by the Editor-in-Chief of this journal.

## Competing interests

The authors declare that they have no competing interests.

## Authors' contributions

KM helped to make the histopathological and immunohistochemical diagnosis, conceived the study, and helped to prepare the manuscript. RL worked up the clinical details and helped to prepare the manuscript. UM helped to make the histopathological and immunohistochemical diagnosis. All authors read and approved the final manuscript.
